# Difficulties in Eliminating Measles and Controlling Rubella and Mumps: A Cross-Sectional Study of a First Measles and Rubella Vaccination and a Second Measles, Mumps, and Rubella Vaccination

**DOI:** 10.1371/journal.pone.0089361

**Published:** 2014-02-20

**Authors:** Zhifang Wang, Rui Yan, Hanqing He, Qian Li, Guohua Chen, Shengxu Yang, Enfu Chen

**Affiliations:** 1 Zhejiang Provincial Center for Disease Control and Prevention, Hangzhou, Zhejiang Province, P. R. China; 2 Cixi City Center for Disease Control and Prevention, Cixi, Ningbo, P. R. China; 3 Sanmen County Center for Disease Control and Prevention, Sanmen, Taizhou, P. R. China; The Australian National University, Australia

## Abstract

**Background:**

The reported coverage of the measles–rubella (MR) or measles–mumps–rubella (MMR) vaccine is greater than 99.0% in Zhejiang province. However, the incidence of measles, mumps, and rubella remains high. In this study, we assessed MMR seropositivity and disease distribution by age on the basis of the current vaccination program, wherein the first dose of MR is administered at 8 months and the second dose of MMR is administered at 18–24 months.

**Methods:**

Cross-sectional serological surveys of MMR antibodies were conducted by collecting epidemiological data in Zhejiang province, China in 2011. In total, 1015 participants were randomly selected from two surveillance sites. Serum MMR-specific immunoglobulin G levels were tested by enzyme-linked immunosorbent assay. The geometric mean titers and seroprevalence with 95% confidence intervals (CIs) were calculated by age and gender. Proportions of different dose of vaccine by age by vaccine were also identified. Statistically significant differences between categories were assessed by the Chi-square test.

**Results:**

Over 95% seroprevalence rates of measles were seen in all age groups except <7 months infants. Children aged 5–9 years were shown lower seropositivity rates of mumps while elder adolescences and young adults were presented lower rubella seroprevalence. Especially, rubella seropositivity was significantly lower in female adults than in male. Nine measles cases were unvaccinated or unknown vaccination history. Among them, 66.67% (6/9) patients were aged 20–29 years while 33.33% (3/9) were infants aged 8–12 months. In addition, 57.75% (648/1122) patients with mumps were children aged 5–9 years, and 50.54% (94/186) rubella cases were aged 15–39 years.

**Conclusions:**

A timely two-dose MMR vaccination schedule is recommended, with the first dose at 8 months and the second dose at 18–24 months. An MR vaccination speed-up campaign may be necessary for elder adolescents and young adults, particularly young females.

## Introduction

Measles, mumps, and rubella are viral infections that are preventable through vaccination programs. Under a national Expanded Program on Immunization (EPI), a one-dose, single-antigen, live attenuated measles vaccine (MV) was used in a limited population aged 8 months for a short period in Zhejiang province, China between the late 1970s and early 1980s. In 1985, the MV program was amended so that an additional dose could be administered at 7 years of age. This schedule was modified again in 2007, with the MV being replaced by a routine measles-containing vaccination providing a measles–rubella vaccine (MRV) at 8 months of age, followed by a measles–mumps–rubella (MMR) vaccine at 18–24 months of age. Since 2008, revaccination policy has been implemented with MRV for the secondary school students. In 2010, Supplementary Immunization Activity (SIA) was achieved throughout the whole country. This large-scale measles vaccination campaign was held on September, 2010, with providing a measles-mumps vaccine (MMV) to children aged from 8 months to 4 years old in the province. However, despite the safe, free, and high uptake rate of the two doses of measles-containing vaccine (MCV) and rubella-containing vaccine (RCV) and one dose of mumps-containing vaccine (MuCV), measles, mumps, and rubella remain common diseases throughout Zhejiang province. Measles outbreaks continued in 2008, with 12782 cases reported, which translated to 252.61 per million of the population. From 2009 to 2011, the incidence of measles remained high at 3.14–17.2 per million of the population. Similarly, the incidence of mumps increased from 394.32 to 558.26 per million of the population in 2007 and 2008, respectively. Finally, the reported cases of rubella increased from 3284 to 4284 in 2007 and 2011, respectively, representing a 30.45% increase or an increase from 65.94 to 78.71 per million of the population. Therefore, the elimination of measles and control of mumps and rubella are urgent public health priorities in local regions. Serological surveillance can be effective in achieving these goals [1], [2].

In our study, we determined the incidence, seroprevalence and vaccination history of MMR in Zhejiang Province in 2011 to clarify the population immunity characteristics and aid in the development of improved vaccination strategies.

## Methods

### Study subjects

A population-based cross-sectional surveillance study was conducted at two surveillance sites (Sanmen county and Cixi city) in healthy population in Zhejiang Province between June and December 2011. The total of 16 towns within Sanmen county and 20 within Cixi city were stratified into 5 regions (east, west, north, south, and center), respectively. The 5 towns in each site were sampled from each region at random. At least 60 individuals within each selected towns were systematically sampled from the inhabitants register to be representative by age and gender.

According to the policies and conventions on routine obligatory vaccination provided by the Ministry of Health of China in 2005, the sample size required to determine population immunity should be 30–50 per age group per surveillance site. Our study assessed 10 age groups: 0–7 months, 8–12 months, 2–4 years, 5–9 years, 10–14 years, 15–19 years, 20–29 years, 30–39 years, 40–49 years, and ≥50 years. In total, at least 300 study subjects were randomly chosen from each surveillance site, with approximately 30 participants randomly selected from each age group of each site.

Eligible subjects were selected from the two sites where they had consistently lived for at least 6 months. Participants were excluded if they had any acute disease or immunodeficiency, or had a history of disease of immune system, or had a history of using immunosuppressive agents. Study subjects were also ineligible if they had a previously received blood products or immunoglobulin during the recent 3 months.

Vaccination status was determined by checking immunization record book for those subjects aged younger than 15 years old and by recalling for others who don't have immunization card. Disease status was confirmed by laboratory diagnosis or physician clinical diagnosis. The ethics committee of Zhejiang provincial center for disease control and prevention approved all study materials, including the study protocol and written materials provided to the subjects, parents, or legal guardians. Written informed consent was obtained from all participants, parents, or legal guardians.

### Antibody assay

A 3–5-ml blood sample was obtained via the median cubital vein, immediately centrifuged, and transferred into polypropylene tubes for storage at −20°C. Serological tests were performed at the measles laboratory of the Department of Expanded Program on Immunization, Zhejiang Provincial Center for Disease Control and Prevention. This Laboratory meets the accreditation criteria for WHO National Measles Laboratories. Immunoglobulin G (IgG) antibodies against measles, mumps, and rubella were measured in the sera by enzyme-linked immunosorbent assay (ELISA) using specific commercially available kits (Virion/Serion GmbH, Germany). All samples were determined with the kits of the same lot number. The control and standard sera were ready to use without further dilution. For each test run, control and standard sera were included independent of the number of microtest strips used. The standard sera were set up in duplicate. All samples were rigorously measured according to the manufacturer's instructions and the results were expressed quantitatively.

A measles IgG antibody concentration of >200 mIU/ml was considered positive, a concentration of <150 was negative while a concentration of between 150 and 200 was equivocal. Mumps IgG antibody concentrations greater than 100 U/ml were detected as positivity. Mumps antibody values less than 70 U/ml and 70–100 were negative and equivocal, respectively. Concentration of samples between 70 and 100 was equivocal. Rubella seropositivity was defined as a titer of >20 IU/ml, a titer of <10 was seronegative, and a titer between 10 and 20 was equivocal. Sera with equivocal results were retested.

### Source of disease information

Data on the incidence and age distribution of measles, mumps, and rubella were obtained from the National Electronic Disease Surveillance System (NEDSS) of China.

### Statistical analysis

The geometric mean titers (GMTs) and antibody seroprevalence were calculated with 95% confidence intervals (CIs). GMTs were calculated using log-transformed individual concentrations and were reported as back-transformed titers. The seropositive prevalence among different groups was compared using the chi-square test. Statistical significance was considered when *P* was *≤*0.05. Data analysis was performed using SPSS, version13.0 (SPSS Inc., Chicago, IL). Graphs were produced with Microsoft Office Excel 2007.

## Results

### Baseline demographics of subjects from the two surveillance sites

We enrolled 1015 subjects in 2011, of which 87 (8.6%) subjects had developed measles, 1 (0.1%) had developed rubella, and 19 (1.9%) had developed mumps during the previous year. Table 1 shows the mean age, age range, and gender distribution by age. Moreover, the changing MCV vaccination strategies under EPI are also listed by age group in Table 1. Vaccination policy was varied with years based on national recommendation of Ministry of Health of China, the various disease burden, health priorities and financial capacity as well.

**Table 1:** Characteristics of subjects in the two surveillance sites.

Characteristics	Male	Female	Total (%)	MCV in EPI	SIA
Mean age ± SD (ys)	20.40±19.80	25.96±20.03	26.41±20.07		
Age range (ys)	0–81.00	0–79.00	0–81.00		
0m–7ms	70	60	130 (12.8)	NA	
8ms–1y	34	24	58 (5.7)	MR1	MMV in 2010
2ys–4ys	37	27	64 (6.3)	MR1 and MMR2	MMV in 2010
5ys–9ys	55	45	100 (9.9)	MV1 and MV2	
10ys–14ys	25	35	60 (5.9)	MV1 and MV2	
15ys–19ys	36	31	67 (6.6)	MV1 and MV2	MRV since 2008
20ys–29ys	47	96	143 (14.1)	MV1 and MV2	
30ys–39ys	66	86	152 (15.0)	Limited number of MV given	
40ys–49ys	56	68	124(12.2)	Vaccine unavailable	
≥50 ys–	41	76	117(11.5)	Vaccine unavailable	
Total	467	548	1015(100)		

m,month; ms, months; yr, year; ys, years; NA, Not Applicable; MCV, measles-containing vaccine; MR1, the first dose of measles-rubella vaccine; MMR2, the second dose of measles-mumps-rubella vaccine; MV1, the first dose of measles vaccine; MV2: the second dose of measles vaccine; MMV, measles-mumps vaccine; MRV, measles-rubella vaccine; SIA, Supplementary Immunization Activities.

### Reported cases of measles, mumps, and rubella at the two surveillance sites in 2011

**Figure 1:**
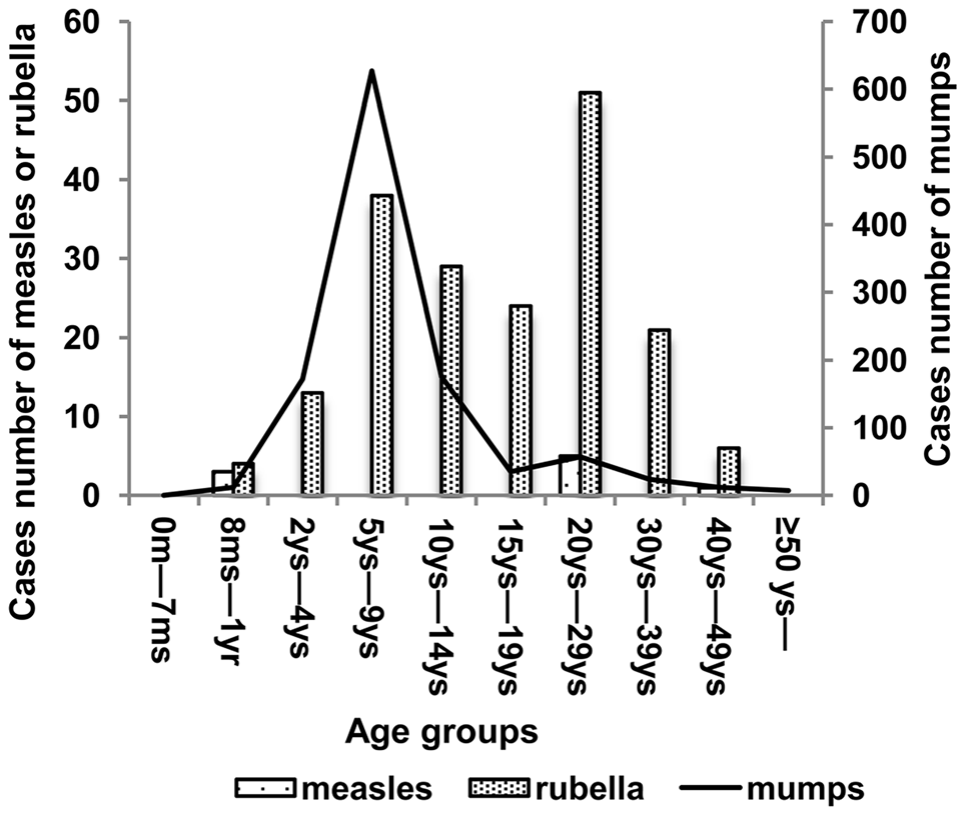
Reported cases number of measles, mumps and rubella by age group in 2011.

The incidence of measles, mumps, and rubella was 10.61, 648.14, and 93.23 per one million inhabitants, corresponding to overall reported cases of 9, 1122, and 167, respectively. Figure 1 (also see Table S1, Table S2 and Table S3) presents the reported number of cases of measles, mumps, and rubella by age group in 2011. Of the 9 patients with measles, 3 (33.33%) were infants aged 8–12 months who were not yet immunized, while 6 (66.67%) were young adults aged 20–29 years who were unsure of their vaccination histories. Of the 1122 patients with mumps, 648 (57.75%) were children aged 5–9 years. Among the 186 patients with rubella, 94 (50.54%) were aged 15–39 years, while 38 (20.43%) were children aged 5–9 years. Vaccination history data was unavailable for patients with mumps and rubella.

### Vaccination history by age at the two surveillance sites in 2011

**Figure 2:**
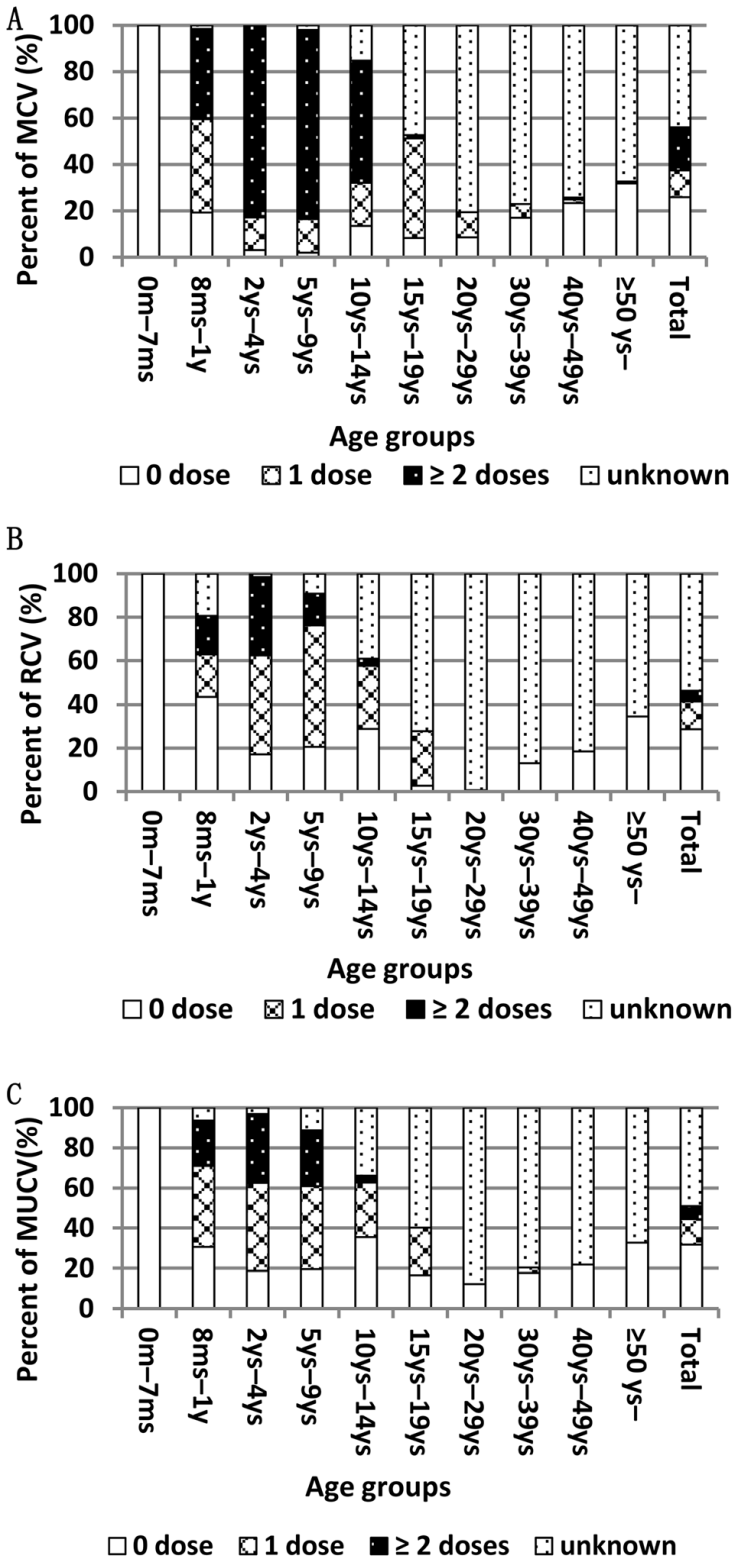
Percentage of vaccination history with different dose by age in 2011. Approximately 90% of children aged <14 years old received at least one dose of MCV and 70% was immunized with two doses (A). However, adults aged >15 years reported with lower coverage of RCV (B) and less than one half of children aged 5–9 years were recorded with two dose of MuCV (C). MCV: measles-containing vaccine; RCV: rubella-containing vaccine; MuCV: mumps-containing vaccine.

Figure 2 (also see Table S1, Table S2 and Table S3) shows proportion of vaccination history of different dose of MCV, RCV, or MuCV by age at these two surveillance points in 2011. At least one dose of MCV were administered to 80% of infants aged 8 months–1 year, 97% of children aged 2–4 years, and 96% of those aged 5–9, respectively. Approximately 85% of children aged 2 years–4 years and 81% of those aged 5–9 years received two doses of MCV. About 70% of children aged 2 years–9 years were given at least one dose of RCV or at least one dose of MuCV. Nevertheless, less than 30% of adults aged 15 years–39 years received one dose of RCV and no more than 40% of children aged 5–9 were immunized with two doses of MuCV.

### Seroprevalence and GMTs of measles, mumps, and rubella antibodies in the different age groups

Seropositivities for measles, mumps, and rubella by age are shown in Table 2.

**Table 2:** Seroprevalence by age group in two surveillance sites.

	Measles (%)	Mumps (%)	Rubella (%)
Age groups	Positive rate (95%CI)	Positive rate (95%CI)	Positive rate (95%CI)
0m–7ms	71.5 (63.7–79.3)	63.1 (54.8–71.4)	54.6 (46.0–63.2)
8ms–1y	98.3 (95.0–100.0)	60.3 (47.7–72.9)	86.2 (77.3–95.1)
2ys–4ys	95.3 (90.1–100.0)	92.2 (85.6–98.8)	93.8 (87.9–99.7)
5ys–9ys	99.0 (97.0–100.0)	86.0 (79.2–92.8)	80.0 (72.2–87.8)
10ys–14ys	98.3 (95.0–100.0)	91.7 (84.7–98.7)	71.7 (60.3–83.1)
15ys–19ys	95.5 (90.5–100.0)	94.0 (88.3–99.7)	79.1 (69.4–88.8)
20ys–29ys	96.5 (93.5–99.5)	95.8 (92.5–99.1)	82.5 (76.3–88.7)
30ys–39ys	96.1 (93.0–99.2)	90.1 (85.4–94.8)	78.9 (72.4–85.4)
40ys–49ys	96.8 (93.7–99.9)	93.5 (89.2–97.8)	70.2 (62.1–78.3)
≥50 ys–	96.6 (93.3–99.9)	94.0 (89.7–98.3)	64.1 (55.4–72.8)

m,month; ms, months; yr, year; ys, years; CI, confidence interval.

The overall seroprevalence of measles was 93.6% in the 1015 participants, with a GMT of 1109.21±2.77 mIU/ml. Seropositivity increased from 71.5% among infants aged <7 months old to 98.3% among those aged 8–12 months old; the majority of children (98%) aged between 8 months and 2 years had received at least one dose of the MCV. Seropositivity levels decreased slightly at 2–4 years of age and gradually increased to 99.0% in the ≥10-year age group, where it remained in the range of 95.5%–99.0%.

The overall seropositivity of mumps was 86.7%, with a GMT of 342.34±2.98 U/ml. Before vaccination, seropositivity remained low from birth to 1 year of age. At the age of 2 years, the coverage of the mumps-containing vaccine was 97%. Therefore, seropositivity abruptly increased to 92.2% in the 2–4-year group, decreased to 86.0% in the 5–9-year group, and fluctuated in the range of 90.1%–95.8% in the ≥10-year group.

**Figure 3:**
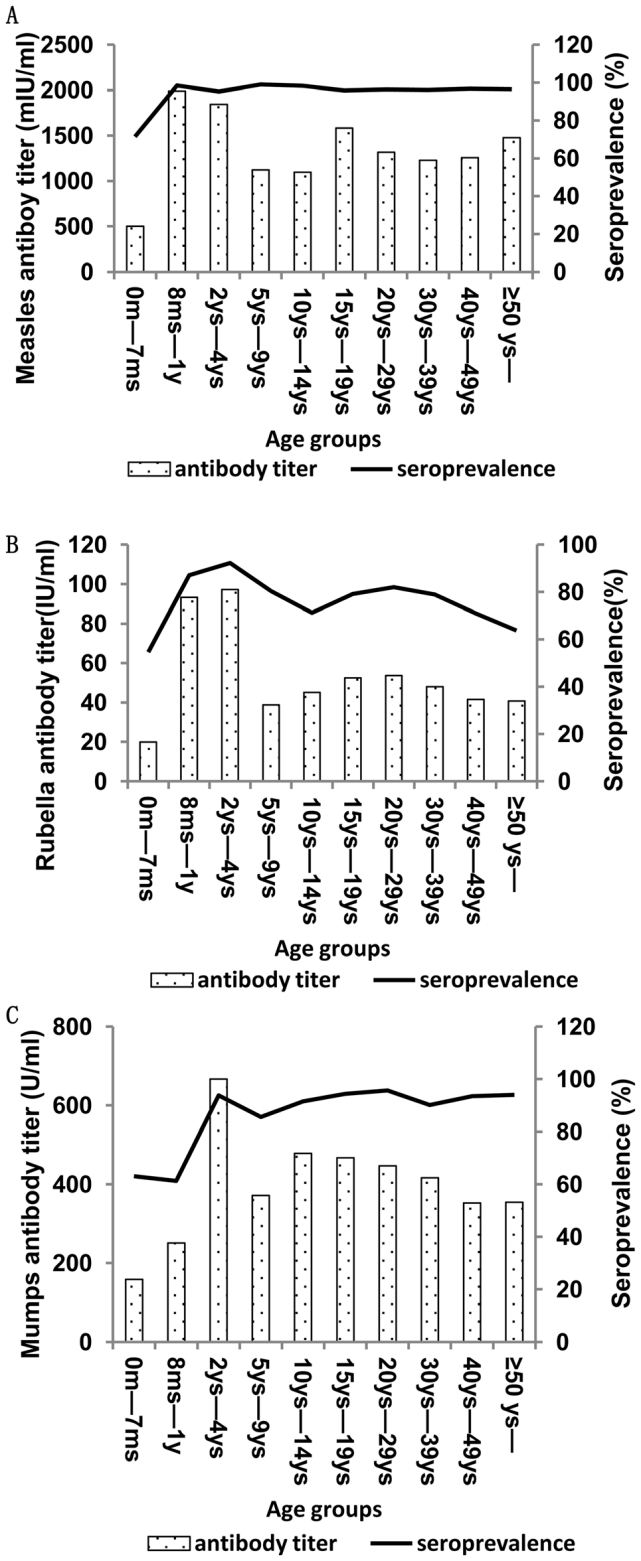
GMTs and seroprevalence of measles, mumps and rubella antibodies in different age group in 2011.

The overall seropositivity of rubella was 74.6%, with a GMT of 42.37±3.26 IU/ml. Seropositivity increased from 54.6% at 0–7 months to a peak of 93.8% at 2–4 years, with 90% coverage of the rubella-containing vaccine during this period. Subsequently, it decreased to 71.7% in the 10–14-year group, increasing to 82.5% in the 20–29-year group and decreasing to 64.1% in the ≥50-year group. The GMTs of measles, mumps, and rubella (Table 3) followed the trend of seropositivity in each age group (Fig. 3).

**Table 3:** GMTs by age group in two surveillance sites.

	Measles (mIU/ml)	Mumps (U/ml)	Rubella (IU/ml)
Age groups	GMT (95%CI)	GMT (95%CI)	GMT (95%CI)
0m–7ms	501.19 (382.20,657.05)	158.49(120.89,207.78)	19.95(14.55,27.37)
8ms–1y	1990.67(1610.21,2461.05)	251.19(159.96,394.46)	93.33(65.04,133.91)
2ys–4ys	1845.02(1526.44,2230.08)	666.81(508.63,874.18)	97.27(94.29,100.35)
5ys–9ys	1122.02(979.94,1284.69)	371.12(291.18,474.07)	38.73(28.49,52.64)
10ys–14ys	1096.48(874.98,1374.04)	478.64(335.09,683.66)	45.08(28.07,72.41)
15ys–19ys	1584.89(1264.74,1986.09)	467.74(373.25,586.14)	52.48(40.03,68.80)
20ys–29ys	1318.26(1130.73,1536.88)	446.68(383.14,520.76)	53.70(42.85,67.30)
30ys–39ys	1230.27(1050.51,1440.79)	416.87(357.57,486.00)	48.08(38.89,59.45)
40ys–49ys	1258.93(1074.98,1474.35)	352.37(303.61,408.96)	41.49(33.41,51.53)
≥50 ys–	1479.11(1234.81,1771.74)	354.81(296.21,425.01)	40.73(31.07,53.41)

m,month; ms, months; yr, year; ys, years; CI, confidence interval; GMT, geometric mean titer.

### Seroprevalence and GMTs of measles, mumps, and rubella antibodies in different vaccination schedules

Measles seropositivity was higher in the participants who had received a second dose of MCV than in those who had received a single dose, while GMTs followed a similar trend as that followed by the seropositive rates. The similar results were found for seropositive rates of mumps or rubella if more than two doses of MuCV or RCV were administered compared with one dose (data not shown). Seropositivity rates for one-dose and two-dose regimes were higher for measles-specific antibodies but lower for rubella-specific antibodies.

### Seroprevalence and GMTs of measles, mumps, and rubella antibodies in males and females

Gender differences were observed for rubella and measles in the 20–29-year and 30–39-year age group, respectively. Seropositivities of measles and rubella were significantly lower in adult females than in adult males (χ^2^_measles_  = 4.794, P = 0.029; χ^2^_rubella_  = 5.443, P = 0.020). No significant gender differences existed in the other age groups (Table 4).

**Table 4:** Seropositivity rates of measles-, mumps- and rubella-IgG antibodies by age and sex in 2011.

	Measles (%)		Mumps (%)		Rubella (%)	
Age groups	Male	Female	Male	Female	Male	Female
0m–7ms	70.0	73.3	55.7	71.7	50.0	60.0
8ms–1yr	100.0	95.8	52.9	70.8	91.2	79.2
2ys–4ys	97.3	92.6	94.6	88.9	91.9	96.3
5ys–9ys	100.0	97.8	85.5	86.7	85.5	73.3
10ys–14ys	100.0	97.1	96.0	88.6	64.0	77.1
15ys–19ys	97.2	93.5	91.7	96.8	83.3	74.2
20ys–29ys	97.9	95.8	95.7	95.8	91.5	78.1*
30ys–39ys	100.0	93.0*	95.5	86.0	84.8	74.4
40ys–49ys	94.6	98.5	96.4	91.2	76.8	64.7
≥50ys–	95.1	97.4	90.2	96.1	68.3	61.8

m,month; ms, months; yr, year; ys, years;*: P≤0.05.

## Discussion

There are overall five surveillance sites (which were located in Cixi city, Haining city, Xianju county, Sanmen county, and Quzhou city as well, respectively.) in Zhejiang province of China in 2011. IgG antibodies against measles, mumps and rubella in the sera of healthy population in local areas were tested in these five disease surveillance points. Only two surveillance sites (both Ci'xi city and Sanmen county) were chosen by our study due to the poor compliance of other three surveillance sites. Thus, the sampling surveillance sites constitute a limitation in our study when it was assumed to represent the whole province. However, it should be ideal to be a representative data from these two surveillance sites.

We conducted a vaccination coverage and serosurvey of IgG antibodies against measles, mumps, and rubella in Zhejiang province, China in 2011. Our results showed that the seropositivity rate in every age group (except 0–7 months) was >95%, even though at least one dose of MCV coverage in the age groups of 10–14 years, 15–19 years, 20–29 years, 30–39 years, 40–49 years, and ≥50 years were reported to be less than 95%, and two-dose MCV coverage was determined to be <95% in most of the age groups except 2–4 years and 5–9 years. It indicted higher level of seroprevalence for measles possibly has been achieved by measles campaigns. Progress toward the goal of WHO measles elimination has been made via both routine and mass vaccination campaigns in the local areas. In 2005, the Regional Committee of WHO Western Pacific Region established 2012 as the target date for regional measles elimination. In 2006, China set a goal of accelerating the progress of eliminating measles by 2012, keeping measles incidence below 0.1 per 100,000, and then developed a series of vaccination strategies. For example, except strengthening two-dose routine measles immunization schedule administered at 8 and 18 months of age, large-scale of SIAs were also included. Likewise, province-wide measles SIAs have been implemented annually for the secondary school students since 2008. Until 2011, approximately 0.5 million teenage students at school were given the MRV each year and the reported vaccine coverage was more than 95%. In addition, to reach the measles elimination goal, China conducted a nationwide measles SIA in September 2010, which is the largest measles campaign in the whole world. In this activity, the target age group was children aged from 8 months to 4 years throughout the whole province and the vaccine was given by MMV. All the children in the age groups were targeted, regardless of resident status or vaccination or disease history. A total of 78551 children were vaccinated through this effort in the two sites and the administrative vaccination coverage of measles vaccine was also over 95%. The subsequent measles cases are at an historic low from 328 in 2008 to 9 in 2011 and measles incidence decreased markedly to 1.061 per 100,000 in 2011, down from 19.11 per 100,000 in 2008. All these efforts to reach more children and teenagers with MCV have rapidly reduced measles cases in this area between 2008 and 2011, which is promising to achieve the goal (measles incidence <5 per million in 2015) suggested by WHO Strategic Advisory Group of Expert (SAGE) in 2010.

Of note, we determined that measles seropositivity was the lowest (71.5%) in the 0–7-month age group, which is probably related to the fact that this age group is not eligible for being vaccinated under the current vaccination schedule and is assumed to be protected by maternal antibody. Furthermore, this study demonstrated that the maximum cases of measles were found in the 20–29-year age group, followed by the 8–12-month age group with no documented prior doses of MCV. These findings have potential explanations. First, young infants are at a high risk of infection because a proportion of them may not have received the MCV vaccination in time. Second, the measles vaccine was introduced free of cost in China in 1978. Although its coverage has gradually increased with a commensurate reduction in the rate of measles, there remains a potential for increased susceptibility in individuals in their twenties. Further investigation of the case-based reporting system from NEDSS of China found that, in 2011, nine measles cases were unvaccinated or unknown vaccination history, suggesting more catch-up vaccination need to target unvaccinated children and young adults to fill the immunization gaps. Meanwhile, it also showed that two infants among nine measles cases were local residents while the other seven cases (one was infants and the left six were young adults) were all from the other parts of China, indicating that imported measles pose threat to eliminate disease and improving MCV coverage in child and younger adult interprovincial migrates is essential. In developed countries, the incidence of measles in adults became a focus following its control in younger children [3], [4]. After extensive use of MCV in Guangzhou city [2], Jiangsu province [1], Wenzhou city [5], and Hangzhou city [6] in China, the proportion of young adults affected by measles increased significantly. It indicted that, with MCV coverage is enhanced, this age-specific proportion of measles has been shifted from children in period of “control and accelerated control” to infants of <1 year and adults of >15 years in that of “measles elimination”. This phenomenon could be potentially explained as an inevitable period to be close to elimination by strengthening two-dose MCV routine immunization and implementing SIAs. Before the introduction of the effective measles vaccine in 1966, nearly each adult aged over 40 years probably experienced natural measles infection in their childhood [7]. Immunity following natural infection is believed to be lifelong [8], and it is likely that this accounts for the low proportion of measles cases in the ≥40-year age group.

In our study, the overall seropositivity of mumps was lower than the herd immunity threshold of 88%–92% [9]. Even though there is a reported higher MMR coverage, the number of patients with mumps did not significantly decrease in 2011, supporting the view that vulnerability accumulated in those with a low seroprevalence of the mumps antibody. The 5–9-year-old population was primarily affected and exhibited lower seropositivity. Most of them had received a single opportunity to receive the one-dose mumps-containing vaccine under the current vaccination schedule. Therefore, this one-dose immunization schedule has been insufficient in controlling the transmission of mumps, consistent with the global experience [10], [11]. In children aged 8 months, the MMR vaccine may be a better alternative to either the measles or the measles–rubella vaccine, allowing an additional opportunity for every pupil to receive two MMR doses before starting school.

We determined that rubella vaccination coverage was lower than MCV and MuCV coverage, with rubella primarily occurring in the 15–39-year age group. Indeed, the potential for decreased rubella coverage caused by introduction of the MR vaccine under the EPI in 2008 may result in an epidemiological shift of disease incidence to this age group [12]. These results are consistent with those of previous studies conducted in China [13], [14], Germany [15], America [16], and Japan [17]. We determined that the rubella seropositive rates in older adolescents and young adults were lower. Moreover, this rate in females aged 20–29 years was significantly lower than that in males of the same age. This is an important finding because it potentially increases the risk of miscarriage, fetal death, or the congenital rubella syndrome (CRS) in future pregnancies [18], with increased social and personal burdens. Therefore, vaccination strategies on RCV are needed to fill this immunity gap.

The vaccination strategy that utilizes a second MMR dose resulted in higher seroprevalence compared with single-dose regimens, indicating that this would be more effective in the elimination of measles, mumps, and rubella in future strategies. According to the results of this research, there is an urgent need to modify the current MCV program in order to eliminate measles and control mumps and rubella. Specifically, we recommend the following measures. First, the current MMR program should be modified to a two-dose schedule, with one dose at 8 months and a second dose at 18–24 months. Second, more efforts should be made to ensure that 8-month-old infants receive timely MCV. Third, MR speed-up campaigns that focus on older adolescents and young adults, particularly females, should be introduced.

## Supporting Information

Table S1 **Vaccination coverage and reported number of cases for measles by age group.**
(DOCX)

Table S2 **Vaccination coverage and reported number of cases for mumps by age group.**
(DOCX)

Table S3 **Vaccination coverage and reported number of cases for rubella by age group.**
(DOCX)
