# Supplementary material for: Difficulties in Eliminating Measles and Controlling Rubella and Mumps: A Cross-Sectional Study of a First Measles and Rubella Vaccination and a Second Measles, Mumps, and Rubella Vaccination
Source: PLoS One. 2014 Feb 20;9(2):e89361. doi: 10.1371/journal.pone.0089361 (PMC3930734; doi:10.1371/journal.pone.0089361)
Supplement: Table S1 — Vaccination coverage and reported number of cases for measles by age group. (DOCX) [file pone.0089361.s001.docx]

Table S1. Vaccination coverage and reported number of cases for measles by age

|  |  | | MCV (%) | | | |  | Cases |
| --- | --- | --- | --- | --- | --- | --- | --- | --- |
| Age groups | 0 dose | 1 dose | | ≥1dose | ≥2 doses | unknown | Number | Ratio (%) |
| 0m–7ms | 66.92 | 0 | | 0 | 0 | 33.08 | 0 | 0 |
| 8ms–1y | 0 | 19.35 | | 98.39 | 79.03 | 1.613 | 1 | 11.11 |
| 2ys–4ys | 0 | 3.13 | | 100 | 96.88 | 0 | 2 | 22.22 |
| 5ys–9ys | 0 | 2.06 | | 97.94 | 95.88 | 2.06 | 0 | 0 |
| 10ys–14ys | 0 | 13.56 | | 84.75 | 71.19 | 15.25 | 0 | 0 |
| 15ys–19ys | 1.39 | 6.94 | | 51.39 | 44.44 | 47.22 | 0 | 0 |
| 20ys–29ys | 1.44 | 7.19 | | 17.99 | 10.79 | 80.58 | 6 | 66.67 |
| 30ys–39ys | 3.95 | 13.16 | | 19.08 | 5.92 | 76.98 | 0 | 0 |
| 40ys–49ys | 3.23 | 20.16 | | 22.58 | 2.42 | 74.19 | 0 | 0 |
| ≥50 ys– | 5.17 | 26.72 | | 27.59 | 0.86 | 67.24 | 0 | 0 |
| Total | 10.44 | 11.33 | | 41.48 | 30.15 | 48.08 | 9 | 100 |

MCV: measles-containing vaccine
