# Supplementary material for: Difficulties in Eliminating Measles and Controlling Rubella and Mumps: A Cross-Sectional Study of a First Measles and Rubella Vaccination and a Second Measles, Mumps, and Rubella Vaccination
Source: PLoS One. 2014 Feb 20;9(2):e89361. doi: 10.1371/journal.pone.0089361 (PMC3930734; doi:10.1371/journal.pone.0089361)
Supplement: Table S2 — Vaccination coverage and reported number of cases for mumps by age group. (DOCX) [file pone.0089361.s002.docx]

Table S2. Vaccination coverage and reported number of cases for rubella by age

|  |  | | RCV (%) | | | | Cases | |
| --- | --- | --- | --- | --- | --- | --- | --- | --- |
| Age groups | 0 dose | 1 dose | | ≥1dose | ≥2 doses | unknown | Number | Ratio (%) |
| 0m–7ms | 100 | 0 | | 0 | 0 | 0 | 6 | 3.59 |
| 8ms–1y | 43.55 | 19.35 | | 37.10 | 17.74 | 19.35 | 12 | 7.19 |
| 2ys–4ys | 17.19 | 45.31 | | 81.25 | 35.94 | 1.56 | 30 | 17.97 |
| 5ys–9ys | 20.62 | 55.67 | | 70.10 | 14.43 | 9.28 | 20 | 11.98 |
| 10ys–14ys | 28.81 | 28.81 | | 32.20 | 3.39 | 38.98 | 23 | 13.77 |
| 15ys–19ys | 2.78 | 25 | | 25 | 0 | 72.22 | 50 | 29.94 |
| 20ys–29ys | 0.72 | 0 | | 0 | 0 | 99.28 | 21 | 12.57 |
| 30ys–39ys | 13.16 | 0 | | 0 | 0 | 86.84 | 5 | 2.99 |
| 40ys–49ys | 18.55 | 0 | | 0 | 0 | 81.45 | 0 | 0 |
| ≥50 ys– | 34.48 | 0 | | 0 | 0 | 65.52 | 167 | 100 |
| Total | 28.67 | 12.81 | | 17.73 | 4.93 | 53. 60 | 0 | 0 |

RCV: rubella-containing vaccine
