# Supplementary material for: Difficulties in Eliminating Measles and Controlling Rubella and Mumps: A Cross-Sectional Study of a First Measles and Rubella Vaccination and a Second Measles, Mumps, and Rubella Vaccination
Source: PLoS One. 2014 Feb 20;9(2):e89361. doi: 10.1371/journal.pone.0089361 (PMC3930734; doi:10.1371/journal.pone.0089361)
Supplement: Table S3 — Vaccination coverage and reported number of cases for rubella by age group. (DOCX) [file pone.0089361.s003.docx]

Table S3. Vaccination coverage and reported number of cases for mumps by age

|  |  | | MuCV (%) | | | | Cases | |
| --- | --- | --- | --- | --- | --- | --- | --- | --- |
| Age groups | 0 dose | 1 dose | | ≥1dose | ≥2 doses | unknown | Number | Ratio (%) |
| 0m–7ms | 100 | 0 | | 0.00 | 0 | 0 | 0 | 0 |
| 8ms–1y | 30.65 | 40.32 | | 62.90 | 22.58 | 6.45 | 13 | 1.159 |
| 2ys–4ys | 18.75 | 43.75 | | 78.13 | 34.38 | 3.13 | 158 | 14.09 |
| 5ys–9ys | 19.59 | 41.24 | | 69.07 | 27.84 | 11.34 | 648 | 57.75 |
| 10ys–14ys | 35.59 | 27.12 | | 30.51 | 3.39 | 33.90 | 168 | 14.97 |
| 15ys–19ys | 16.67 | 23.61 | | 23.61 | 0 | 59.72 | 37 | 3. 30 |
| 20ys–29ys | 12.23 | 0 | | 0.00 | 0 | 87.77 | 56 | 4.99 |
| 30ys–39ys | 17.76 | 2.63 | | 2.63 | 0 | 79.61 | 22 | 1.96 |
| 40ys–49ys | 21.77 | 0 | | 0.00 | 0 | 78.23 | 13 | 1.16 |
| ≥50 ys– | 32.76 | 0 | | 0.00 | 0 | 67.24 | 7 | 0.62 |
| Total | 31.72 | 12.81 | | 19.21 | 6.40 | 49.06 | 1122 | 100 |

MuCV: mumps-containing vaccine
